# The BET Bromodomain Inhibitor I-BET-151 Induces Structural and Functional Alterations of the Heart Mitochondria in Healthy Male Mice and Rats

**DOI:** 10.3390/ijms20071527

**Published:** 2019-03-27

**Authors:** Jérôme Piquereau, Angèle Boet, Christine Péchoux, Fabrice Antigny, Mélanie Lambert, Mélanie Gressette, Benoît Ranchoux, Natalia Gambaryan, Valérie Domergue, Sharon Mumby, David Montani, Ian M. Adcock, Marc Humbert, Anne Garnier, Catherine Rucker-Martin, Frédéric Perros

**Affiliations:** 1 UMR-S 1180, Inserm, Univ. Paris-Sud, Université Paris-Saclay, 92290 Châtenay-Malabry, France; jerome.piquereau@u-psud.fr (J.P.); melanie.gressette@u-psud.fr (M.G.); anne.garnier@u-psud.fr (A.G.); 2 Réanimation des cardiopathies congénitales, Hôpital-Marie-Lannelongue, Univ. Paris-Sud, 92350 Le Plessis-Robinson, France; angele.boet@hotmail.fr; 3 INSERM UMR_S 999, Hôpital Marie Lannelongue, 92350 Le Plessis Robinson, France; antignyfabrice@gmail.com (F.A.); melanie.lambert91@hotmail.fr (M.L.); benoit.ranchoux@gmail.com (B.R.); david.montani@aphp.fr (D.M.); marc.humbert@bct.aphp.fr (M.H.); catherine.rucker-martin@u-psud.fr (C.R.-M.); 4 Univ Paris-Sud, Faculté de Médecine, Université Paris-Saclay, 94270 Le Kremlin Bicêtre, France; 5 Gabi, Inra, AgroparisTech, Université Paris-Saclay, 78350 Jouy-en-Josas, France; christine.longin@inra.fr; 6 Airway Disease Section, National Heart and Lung Institute, Imperial College London, London SW7 2AZ, UK; natachagam@gmail.com (N.G.); s.mumby@imperial.ac.uk (S.M.); ian.adcock@imperial.ac.uk (I.M.A.); 7 Animal Facility, Institut Paris Saclay d’Innovation Thérapeutique (UMS IPSIT), Université Paris-Sud, Université Paris-Saclay, 92290 Châtenay-Malabry, France; valerie.domergue@u-psud.fr; 8 AP-HP, Service de Pneumologie, Hôpital Bicêtre, 94270 Le Kremlin-Bicêtre, France; 9 Hôpital Marie Lannelongue, Recherche médicale, 92350 Le Plessis Robinson, France

**Keywords:** bromodomain and extra-terminal domain, bromodomain and extra-terminal domain inhibitor, mitochondria, heart, cardiotoxicity

## Abstract

The bromodomain and extra-terminal domain family inhibitors (BETi) are a promising new class of anticancer agents. Since numerous anticancer drugs have been correlated to cardiomyopathy, and since BETi can affect non-cancerous tissues, we aimed to investigate in healthy animals any ultrastructural BETi-induced alterations of the heart as compared to skeletal muscle. Male Wistar rats were either treated during 3 weeks with I-BET-151 (2 or 10 mg/kg/day) (W3) or treated for 3 weeks then allowed to recover for another 3 weeks (W6) (3-weeks drug washout). Male C57Bl/6J mice were only treated during 5 days (50 mg/kg/day). We demonstrated the occurrence of ultrastructural alterations and progressive destruction of cardiomyocyte mitochondria after I-BET-151 exposure. Those mitochondrial alterations were cardiac muscle-specific, since the skeletal muscles of exposed animals were similar in ultrastructure presentation to the non-exposed animals. I-BET-151 decreased the respiration rate of heart mitochondria in a dose-dependent manner. At the higher dose, it also decreased mitochondrial mass, as evidenced by reduced right ventricular citrate synthase content. I-BET-151 reduced the right and left ventricular fractional shortening. The concomitant decrease in the velocity-time-integral in both the aorta and the pulmonary artery is also suggestive of an impaired heart function. The possible context-dependent cardiac side effects of these drugs have to be appreciated. Future studies should focus on the basic mechanisms of potential cardiovascular toxicities induced by BETi and strategies to minimize these unexpected complications.

## 1. Introduction

The bromodomain (BRD) and extra-terminal domain (BET) family inhibitors (BETi) are a promising new class of anticancer agents [1]. Indeed, BET proteins mediate protein–protein interaction networks between diverse arrays of partners, and function as mitosis bookmarks, protein scaffolds and chromatin regulators in cellular processes; controlling proliferation and cell cycle progression. BETi are also promising in the treatment of latent virus infections by disrupting transcriptional co-regulators [1].

The BET family includes BRD2, BRD3, BRD4, and BRDT, which are part of a class of proteins called histone readers. BRD2, BRD3 and BRD4 are ubiquitously expressed, whereas BRDT is localized primarily to the testis [2]. BRD-containing proteins selectively bind to acetyl lysine marks placed by histone modifying enzymes (HATs/KATs) [3]. BET binding to acetylated chromatin subsequently activates RNA Pol II-driven transcriptional elongation. Small molecule BET inhibitors prevent binding of BET proteins to acetylated histones and inhibit transcriptional activation of BET target genes [3]. BETi like JQ1 and I-BET-151, a highly specific and potent inhibitor of BRD2/3/4 [4,5], have shown encouraging activity in multiple preclinical models of medulloblastoma [2], prostate cancer [6], NUT midline carcinoma (NMC), T-Cell Acute Lymphoblastic Leukemia (T-ALL) [7], ovarian cancer [8], drug resistant myeloma [9,10], uveal melanoma [11], breast cancer cells [12], hepatocellular carcinoma [13], Ewing sarcoma [14,15], and gastric cancer [16], among others (please see the review from French Cancer Agency [17] for a recent overview of the BETi in preclinical models).

Since numerous anticancer drugs have been correlated to cardiomyopathy [18,19], and since BETi can affect non-cancerous tissues, we aimed to investigate any ultrastructural alterations of the heart induced by BETi in healthy animals. We focused at the mitochondrial mass and function and not on inflammatory-mediated changes or apoptosis-related changes, because we already observed that the cardiotoxicity of two other chemotherapies (imatinib and masitinib) was related to severe cardiac mitochondrial alterations (unpublished data), observations that were consistent with the findings from Kerkelä et al. [20]. Moreover, we did not observe inflammatory infiltrates nor ultrastructural features of apoptosis in our sections [21]. For this reason, we did not study the inflammatory-mediated changes or apoptosis-related changes in the hearts of I-BET-151 treated animals.

We used transmission electron microscopy (TEM) to examine the hearts from mice and rats exposed to I-BET-151 as compared to non-exposed animals. We found ultrastructural alterations in the mitochondria of cardiomyocytes in exposed animals. For this reason, we performed dose-response experiments followed by a drug washout period in rats to address the potential reversibility of the observed side effects induced by I-BET-151. We analyzed the heart ultrastructure by TEM after the treatment and the washout periods. We also assessed the heart function in all groups at three time points (basal, at the end of the treatment, and after the drug washout periods) by echocardiography and carried out mitochondrial functional assays in permeabilized cardiac ventricular fibers upon sacrifice of the animals. In parallel to our investigations in the cardiac muscle, we also performed TEM analysis of the skeletal muscle to assess the potential systemic effects of BRD inhibition.

## 2. Results

### 2.1. I-BET-151 Induces Heart-Specific Ultrastructural Alterations of Mitochondria in Healthy Male Mice and Rats

**Figure 1:**
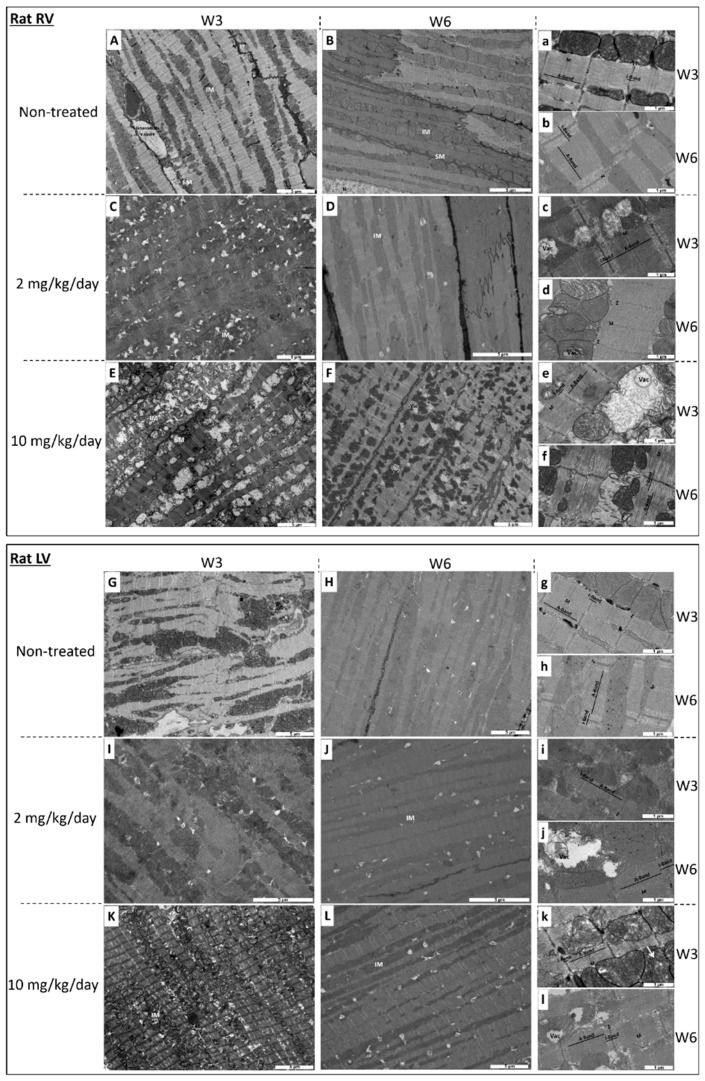
Transmission electron micrograph of right ventricle (RV) and of the left ventricle (LV) in rats at week 3 (W3) (**A**,**a**,**C**,**c**,**E**,**e** for RV and **G**,**g**,**I**,**i**,**K**,**k** for LV) and at week 6 (W6) (3-weeks drug washout) (**B**,**b**,**D**,**d**,**F**,**f** for RV and **H**,**h**,**J**,**j**,**L**,**l** for LV). (**A**,**a**,**B**,**b**,**G**,**g**,**H**,**h**): non-treated rats. (**C**,**c**,**D**,**d**,**I**,**i**,**J**,**j**): 2 mg I-BET-151/kg/day treated rats. (**E**,**e**,**F**,**f**,**K**,**k**,**L**,**l**): 10 mg I-BET-151/kg/day treated rats. SM indicates sub-sarcolemmal mitochondria; IM, interfibrillar mitochondria; Vac, vacuole; Tt, T tubule; M, M line; Z, Z line. Bar scale is indicated on each micrograph. Selection of the most representative pictures from three animals per group.

**Figure 2:**
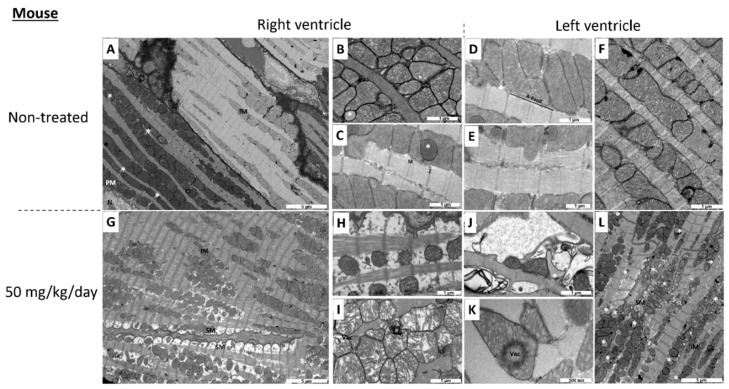
Transmission electron micrograph of non-treated (**A**–**F**) and 50 mg I-BET-151/kg/day treated (**G**–**L**) mouse right ventricle (**A**–**C**,**G**–**I**) and left ventricle (**D**–**F**,**J**–**L**). SM indicates sub-sarcolemmal mitochondria; IM, interfibrillar mitochondria; PM, perinuclear mitochondria, Vac, vacuole; N, nucleus; M, M line; Z, Z line. Asterisks indicate lipid droplets. Bar scale is indicated on each micrograph. Selection of the most representative pictures from three animals per group.

Ultrastructural alterations of the heart tissue are displayed in Figure 1 and Figure 2, and quantified in Table 1.

I-BET-151 led to ultrastructural alterations of heart mitochondria in healthy rats and mice. In control (non-exposed) rats at each stage, mitochondria were aligned along the sarcomeres without intermitochondrial spaces and they were of normal size, i.e., approximately the length of a sarcomere, both in the right (Figure 1A,a,B,b) and in the left (Figure 1G,g,H,h) ventricles. The mitochondrial matrix looked dense and homogeneous with well distributed cristae (i.e., lamellar and parallel organization of the inner membrane), and mitochondrial alterations were very rare (Table 1). Sarcomeres occupied the entire cardiomyocyte area and had a normal structure; the Z-line, and I-band, A-band, and M-line could be easily identified.

**Figure 3:**
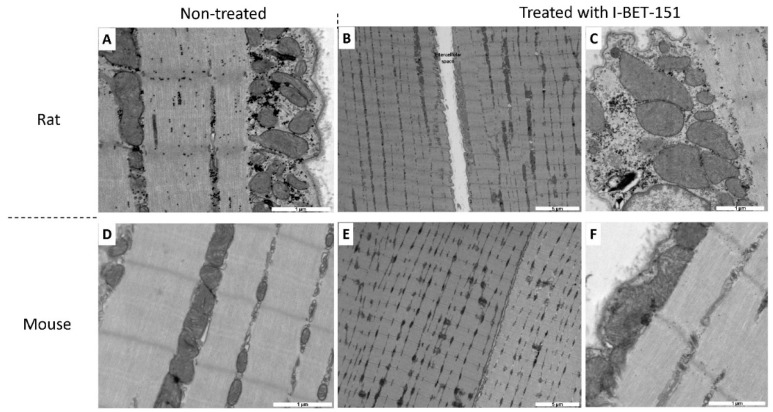
Transmission electron micrograph of non-treated (**A**,**D**) and treated (**B**,**C**,**E**,**F**) rat (**A**–**C**) and mouse (**D**–**F**) skeletal muscle. Rats were treated with 10 mg/kg/day of I-BET-151 while mice were treated with 50 mg/kg/day of I-BET-151. Bar scale is indicated on each micrograph. Selection of the most representative pictures from three animals per group.

After 3 weeks (W3) of exposure with the high dose of I-BET-151 (10 mg/kg/day), we observed a significant alteration of mitochondrial ultrastructure, both in the right (Figure 1E,e) and in the left (Figure 1K,k) ventricles. These alterations affected mitochondria independently of their localization in the cardiomyocyte (i.e., sub-sarcolemmal, interfibrillar, and perinuclear). Mitochondria appeared swollen and more heterogeneous in size and shape. Mitochondria were fragmented with fewer cristae which made the mitochondrial matrix less opaque to electrons. In the right ventricle, this phenotype was accompanied by a complete disorganization of the cristae and vacuolization of the matrix space (i.e., signs of irreversible cell injury). In the left ventricle, some mitochondria had either a rearrangement of the cristae with an “onion ring-like” appearance (not shown), or electron opacification of their content indicating the beginnings of vacuolization (Figure 1k). These mitochondrial alterations were cardiac muscle-specific since skeletal muscles of exposed animals (Figure 3B,C) were similar in ultrastructure to the non-exposed animals (Figure 3A). In contrast, sarcomere ultrastructure was well preserved at W3.

When treated with I-BET 151 at the dose of 10 mg/kg/day, mitochondrial damage seemed less severe in the right ventricle (Figure 1F,f) and seemed to be sustained in left ventricles after the washout period (W6) (Figure 1L,l). Indeed, only 17 ± 4.2% of mitochondrial population exhibited alterations in right ventricle after the washout period while 61.7 ± 10.9% of the mitochondria were damaged at W3 (Table 1). The beneficial effect of this washout period was not observed in the left ventricle in which 61.3 ± 8.4% and 60.9 ± 10.7% of the mitochondria were altered at W3 and W6 respectively (Table 1). However, right ventricular sarcomeres displayed significant I- and A-band alterations at W6 (Figure 1f). These structures were less opaque to electrons suggesting damage of the contractile apparatus.

Although not statistically significant, after 3 weeks of exposure with the low dose of I-BET-151 (2 mg/kg/day), there was a strong trend towards less severe mitochondrial injury as compared to the higher dose, both in the right (Figure 1C,c and Table 1) and in the left (Figure 1I,i and Table 1) ventricles. Even so, these alterations affected mitochondria independently of their localization in the cardiomyocyte. Mitochondrial matrix appeared less opaque to electrons with a disorganization of the cristae and a beginning of vacuolization of the matrix space (i.e., sign of irreversible cell injury) and sarcomere ultrastructure was well preserved. At this low dose, after the washout period (W6) mitochondrial damage seemed irreversible both in the right (Figure 1D,d and Table 1) and in the left ventricles (Figure 1J,j and Table 1) since the mitochondrial modifications and the proportion of altered mitochondria were similar to those observed at W3 (Table 1); on the other hand sarcomeres were preserved. 

In control mice, as is the case in control rats, mitochondria in the right (Figure 2A–C) and in the left (Figure 2D–F) ventricles were numerous and aligned along the sarcomeres without intermitochondrial space. Mitochondria contained well aligned, lamellar and parallel cristae, which extended through the entire body of the organelle with a homogeneous distribution. Sarcomeres occupied the entire cardiomyocyte area and had a normal structure. Some lipid droplets could be observed in the right ventricle (Figure 2B,C). After 5 days of exposure with I-BET-151 (50 mg/kg/day), we observed a significant deterioration of mitochondrial ultrastructure in the right ventricle (Figure 2G–I), which primarily affected sub-sarcolemmal mitochondria. Fewer and smaller mitochondria were observed, and these were characterized by an altered shape, transitioning from a tubular-like to a spherical-like shape. Nonetheless, accurate quantification of these changes was difficult in the absence of 3D reconstruction by tomography. Moreover, the spaces between mitochondria were extensively enlarged (Figure 2H). Some interfibrillar mitochondria had a fragmentation of their cristae making the mitochondrial matrix less opaque to electrons. Sarcomere integrity was altered in the right ventricle, leading to a loss of myofibrils on the periphery of myocytes. In the left ventricle (Figure 2J–L), we observed an abnormal accumulation of lipid droplets suggesting an alteration of the fatty acid metabolism (Figure 2L). Some mitochondria had a total destruction of their inner membrane with loss of cristae (Figure 2J). Others showed signs of vacuolization (Figure 2K). The structure of sarcomeres seemed to be preserved at the exception of some myofibrils. As is the case in rats, I-BET-151 in mice induced cardiac muscle-specific mitochondrial alterations, since the skeletal muscles of exposed animals (Figure 3E,F) were similar in ultrastructure presentation to the non-exposed animals (Figure 3D).

### 2.2. I-BET-151 Decreases Cardiac Mitochondrial Function in Healthy Rats

**Figure 4:**
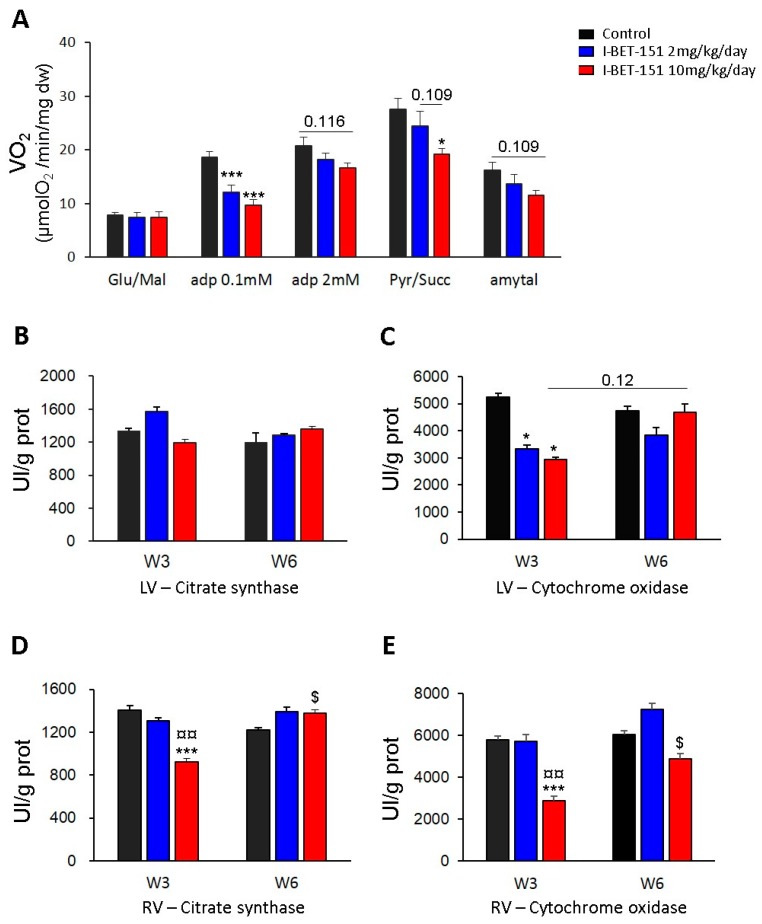
Effect of I-BET-151 on mitochondrial functions. Oxygen consumption rate of permeabilized ventricular fibers with 10 mM glutamate and 4 mM malate, 0.1 mM ADP, 2 mM ADP, 1 mM pyruvate and 15 mM succinate, and after addition of 2 mM amytal (**A**). Citrate synthase activity in left ventricle (**B**). COX activity in left ventricle (**C**). Citrate synthase activity in right ventricle (**D**). COX activity in right ventricle (**E**). W3: Week 3, 3 weeks of exposure to I-BET-151 or vehicle only; Week 6: 3 weeks of exposure to I-BET-151 or vehicle only followed by 3 weeks of drug wash out. Between four and six rats per group. * *p* < 0.05 and *** *p* < 0.001 as compared to control group (at W3 or W6), ^¤^ *p* < 0.05 and ^¤¤^ *p* < 0.01 “10 mg/kg/day group” as compared to “2 mg/kg/day group” (at W3 or W6), ^$^ *p* < 0.05 W3 as compared to W6.

In order to know if changes in mitochondrial structure observed by TEM after I-BET-151 treatment were associated with alterations of mitochondrial function, mitochondrial oxidative capacities were measured in permeabilized fibers prepared from the left ventricle (Figure 4A). In the presence of glutamate and malate (complex I-driven respiration), the respiration rate measured after the addition of 0.1 mM ADP was significantly lower in I-BET-151 treated groups than in the control group. Increased ADP (2 mM), gave a trend (*p* = 0.116) towards a lower maximal complex I-driven respiration in fibers from animals after 3 weeks of treatment with the high dose of I-BET-151 (10 mg/kg/day). This difference in oxygen consumption between control and high dose groups became statistically significant when pyruvate and succinate (allowing the electron transfer from complex II) were added in the respiration solution, suggesting a decreased mitochondrial function in the myocardium of rats treated with 10 m/kg/day of I-BET-151. 

In contrast, three weeks of treatment with low dose of I-BET-151 (2 mg/kg/day) did not significantly affect the mitochondrial oxidative capacities of the rat heart (with 2 mM ADP and pyruvate/succinate). The end of the protocol required addition of amytal (a complex I inhibitor) which also revealed data suggesting deleterious effects of high dose of I-BET-151 treatment. Indeed, a strong trend towards lower respiration rates (complex II-driven respiration) was observed following amytal in the high dose group whilst the effects in fibers from 2 mg/kg/day I-BET-151 treated animals was less marked. 

Although the activity of citrate synthase (CS), which is commonly used as a marker of mitochondrial mass, was not affected in left ventricle at W3 or W6 with low or high dose of I-BET-151 (Figure 4B), the cytochrome oxidase (COX) activity was significantly decreased after 3 weeks of treatment with 2 or 10 mg/kg/day of I-BET-151 (Figure 4C). Surprisingly, three weeks after treatment cessation (W6), this parameter seemed to be completely restored in the high dose group (*p* = 0.12, W3 vs. W6) while in the low dose group, it did not significantly increase between W3 and W6 and tended to stay lower than control group after the washout period although this difference was not statistically significant. The measurement of these same enzyme activities in the right cardiac ventricle (RV) showed significant decreases in these parameters at the end of the treatment (W3) with I-BET-151 at the dose of 10 mg/kg/day (Figure 4D,E); these activities were significantly restored after three weeks of washout (W6). The activity of these enzymes in the right ventricle was not altered by treatment with the low dose of I-BET-151.

Overall, these data indicate that I-BET-151 altered mitochondrial function in a dose-dependent manner inasmuch as the effects of the low dose treatment were much less marked than the effects observed in the high dose group. In this latter case, the fact that the decreases in oxygen consumption and COX activity at W3 were associated with no alteration in CS activity strongly suggests that the treatment specifically affects the mitochondrial respiratory chain without having any consequences on mitochondrial mass. Although the alterations of mitochondrial functions are not as clear in the low dose group as they are in the high dose group, the decrease in COX activity in this group at W3 demonstrates mitochondrial damage. Nevertheless, the consequences on maximal mitochondrial respiration (complex I-driven respiration and complex-II driven respiration) were not clearly shown since COX is not a limiting enzyme and because the mitochondrial damage induced by low dose treatment was too mild to induce major damage to mitochondrial respiration. Yet, the significant decrease in respiration rate measured with 0.1 mM ADP in both treated groups proved that cardiac mitochondria of rats treated with 2 mg/kg/day are not identical to fully healthy mitochondria of the control rat hearts. Although the decrease in these parameters only suggests changes in ADP affinity of the mitochondria, this could imply important reorganization of these organelles. The fact that COX activity recovery in LV at W6 (three weeks after having stopped the treatment) was not as clear in low dose group as it was in high dose group has to be considered and could mean that even if the mitochondria only seem to be mildly affected, the damages could be irreversible.

### 2.3. I-BET-151-Induced Ultrastructural and Metabolic Alterations of Cardiac Mitochondria Affect Heart Function

**Figure 5:**
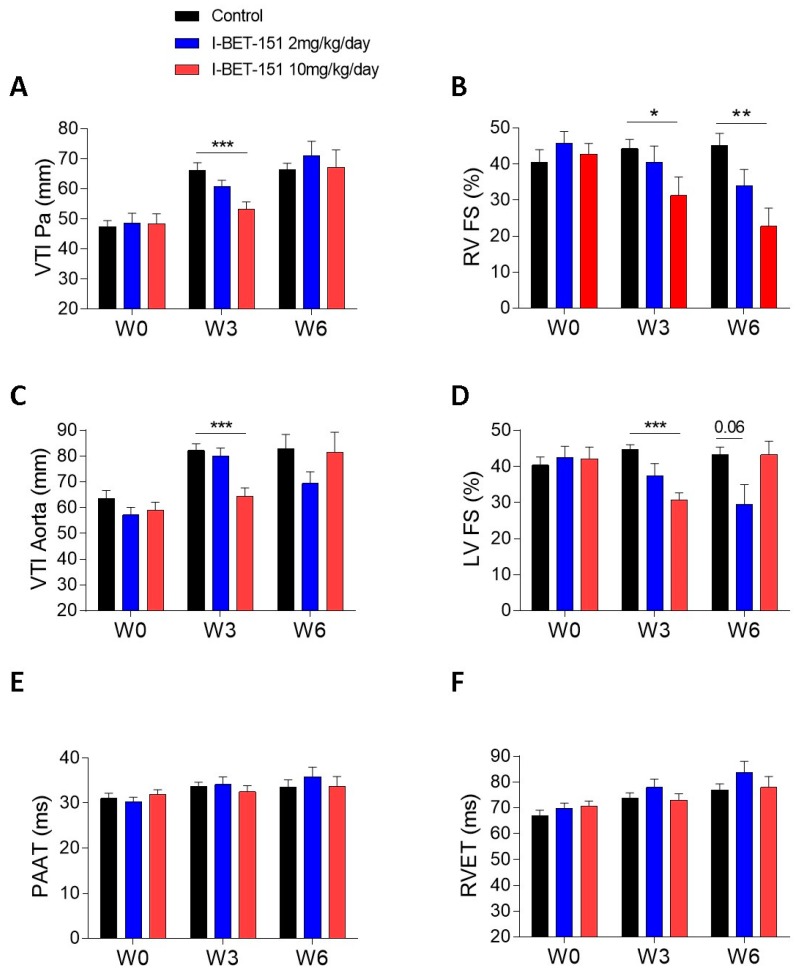
Effect of I-BET-151 on heart function assessed by echocardiography. Velocity-time-integral (mm) in the pulmonary artery (**A**). Right ventricular fractional shortening (%) (**B**). Velocity-time-integral in the aorta (mm) (**C**). Left ventricular fractional shortening (%) (**D**). Pulmonary artery acceleration time (ms) (**E**). Right ventricular ejection time (ms) (**F**). Between six and 12 rats per group (12 at baseline and W3, six at W6). W0: baseline, W3: Week 3, 3 weeks of exposure to I-BET-151 or vehicle only; Week 6: 3 weeks of exposure to I-BET-151 or vehicle only followed by 3 weeks of drug wash out. * *p* < 0.05, ** *p* < 0.01, and *** *p* < 0.01 as compared to control group (at W0, W3, or W6).

At the high dose (10 mg/kg/day I-BET-151), TEM analyses showed clear mitochondrial damage that seemed less severe after the washout period, especially in RV (Figure 1F,L,l and Table 1). The metabolic assays demonstrated a patent decrease in oxidative capacities of cardiac mitochondria after this high dose treatment (Figure 4A). Enzyme activity measurements in high dose groups supported this finding (decrease in the COX activity in the LV and in the CS and in the COX activities in the RV) and demonstrated a real recovery after the washout period since COX and CS activity values went back to control values (Figure 4B–E). This correlates with the reduction in the left ventricular fractional shortening values (LVFS, %) after the treatment period and their recovery after the washout period (Figure 5D). By contrast, the persistent (even the progressive) reduction of the right ventricular fractional shortening (RVFS, %) after the washout period (Figure 5B) does not correlate with the metabolic recovery and the normalization of the mitochondrial ultrastructure at this time point. However, the alterations in the contractile apparatus that we found specifically in the RV cardiomyocytes (Figure 1F) may partly explain this discrepancy. The decrease in the velocity-time-integral (VTI) in both the aorta and the pulmonary artery observed at the high dose is also suggestive of an impaired heart function (Figure 5A,B).

At a low dose (2 mg/kg/day I-BET-151), TEM analyses showed less severe mitochondrial injuries as compared to the higher dose (Table 1). Yet, the observed alterations seem irreversible since there was no apparent improvement after drug washout (Figure 1D,d,J,j and Table 1). The biochemical analyses confirmed that the mitochondrial damage was modest at this low dose, since only the COX level was statistically decreased in the left ventricle (LV) (Figure 4C). Metabolic analyses showed a small trend towards a reduction in mitochondrial respiration in this group (Figure 4A). Interestingly, COX levels remained low after the washout period as compared to the high dose treatment although this difference did not reach significance. This suggests that low dose-induced mitochondrial damage, although small, may be irreversible. This fits with the reduced RVFS and LVFS that persisted in this group after drug washout (Figure 5B,D). 

## 3. Discussion

The ultrastructural alterations and progressive destruction of heart muscle mitochondria but not of skeletal muscle mitochondria after I-BET-151 exposure suggest that BET inhibition may have detrimental specific effects on energy generation and/or cardiac function in healthy rats and mice. We provide evidence that I-BET-151 decreases the respiration rate of mitochondria in a dose-dependent manner with complex I- and II-driven respiration being particularly affected. At the higher dose (10 mg/kg/day), it also decreased mitochondrial mass, as evidenced by reduced RV content in citrate synthase. Those modifications may decrease myocardial contractility, as suggested by the significant reductions in RVFS and LVFS. The decrease in the velocity-time-integral (VTI) in both the aorta and the pulmonary artery observed at the same dose is also suggestive of an impaired heart function. I-BET-151-induced functional, metabolic and ultrastructural cardiac alterations were partially reversible after 3 weeks washout.

This observation appears to contradict previous studies demonstrating that BET inhibition alleviates heart failure (HF) in experimental models of pressure overload [22,23,24] and of acute myocardial infarction (AMI) [25]. One explanation for this apparent contradiction is that BRD4 protein expression is increased during the TAC-induced cardiac hypertrophy [22] even though it is not consistently reported [23]. BRD2 and BRD4 mRNA and protein expression levels are also significantly increased in the AMI group compared with those in the sham group [25]. BET inhibition could be beneficial at high BET expression to restore “healthy” BET activity whereas it could be detrimental at basal BET expression as seen here in healthy control rats; leading to a failure of normal BET homeostasic functions. Another possible explanation is that all three studies cited above used JQ1 as the BET inhibitor whereas we used I-BET-151. It may be that the heart ultrastructural alterations we observed are compound- and not class-specific. However, the in vitro comparison of dissimilar BET inhibitors (I-BET, I-BET-151, RVX-208, and PFI-1) demonstrated that at equimolar doses, the inhibition of agonist-induced cardiomyocyte hypertrophy was a class effect of BET inhibitors [23]. Of course, a remaining general limitation associated with conventional occupancy-driven target inhibition is that it often demands full target engagement, requiring sustained high concentrations of a potent small molecule inhibitor over a prolonged time. This in turn enhances off-target effects and can lead to unwanted side effects or toxicity in a therapeutic setting [26].

The potential cardiac toxicity of I-BET-151, this deleterious effect could be mediated through c-Myc antagonism. Indeed, c-Myc inhibitors, including BET inhibitors, share a common mechanism of action involving ATP depletion [27]. This involves the depletion of ATP stores due to mitochondrial dysfunction and the eventual down-regulation of c-Myc protein. Because c-Myc is needed to sustain glycolysis, mitochondrial biogenesis and oxidative phosphorylation [28,29,30], the loss of its function upon inhibitor treatment leads to a rapid suppression of these energy-generating pathways resulting in either terminal differentiation or apoptotic cell death. The effects of c-Myc depletion on ATP levels can be mimicked by pharmacologic inhibition of the mitochondrial electron transport chain without affecting Myc levels [27]. At the ultrastructural level, mitochondrial mass is significantly reduced and the remaining organelles become atrophic in the absence of c-Myc [29,30]. The mitochondria of *c-Myc*^−/−^ cells were not only less abundant and smaller (average length approx. 500 nm) than those of *c-Myc*^+/+^ cells (average length approx. 800 nm), but lacked elaborate cristae patterns [30]. In accordance with the reversibility of the deleterious consequences of Brd4 inhibition [31], reconstitution of c-Myc in *c-Myc*^−/−^ cells, not only partially rescues mitochondrial mass but also increases the number of morphologically normal mitochondria as determined by TEM [29]. From these studies, we could hypothesize that the ultrastructural alterations of heart muscle mitochondria we observed in healthy mice and rat exposed to I-BET-151 may be the consequence of c-Myc inhibition.

Our study has limitations. First, we didn’t analyze the possible gender-specific effect of I-BET-151. This is indeed a relevant issue, as a sexual dimorphism of some anticancer drug-related cardiotoxicities has been described in humans and in animal models such as in anthracycline (doxorubicin)-mediated cardiotoxicity [32]. We have previously reported mitochondrial dysfunction and energy signaling as a critical mediator of sex differences in doxorubicin cardiotoxicity in rats [33]. After 7 weeks of doxorubicin (2 mg/kg/day per week), male Wistar rats developed major signs of cardiomyopathy with cardiac atrophy, reduced left ventricular ejection fraction and 50% mortality. In contrast, no females died and their left ventricular ejection fraction was only moderately affected. Since numerous anticancer drugs have been correlated to cardiomyopathy and heart failure, and since BETi can affect non-cancer tissues, it was important to explore the cardiac side effects of BETi in animals. We decided to first use males in this study since they are more sensitive to anticancer drug-related cardiotoxicity. Males clearly demonstrated alterations in cardiac morphology and function after I-BET-151 exposure and since mitochondria have a central role for sex differences in pathologies [34], the question of a sex-specific effect in BETi-mediated cardiotoxicity has to be addressed. At present, clinical and experimental studies regarding the role of BETi in cancer therapy or their side effects have been performed in groups including both males and females or in male or female animals respectively. There is no data available comparing both sexes. Thus, retrospective and prospective human studies as well as basic studies are needed in order to understand the basis for a possible sexual dimorphism of BETi cardiotoxicity and to develop new therapeutic approaches. Second, the known BRD inhibition-induced gastrointestinal (GI) toxicity [31] may favor cardiac dysfunction. For instance, it is recognized that GI damage may be a critical factor in acute iron poisoning [35]. Loss of body fluid following GI injury may lead to decreased blood volume and increased blood viscosity. Blood pressure and tissue perfusion decrease, leading to diminished cardiac output and eventual cardiac failure. There is also an increasing evidence to suggest that a “leaky” bowel wall may lead to translocation of bacteria and/or endotoxin, which may be an important stimulus for inflammatory cytokine activation in chronic heart failure [36] and in pulmonary arterial hypertension [37]. Third, we did not analyze the glycolysis that could compensate the decrease in the respiration rate of the mitochondria. Nonetheless, the decreased heart function associated with the reduced mitochondrial respiration induced by I-BET-151, is highly suggestive that glycolysis does not compensate the loss of energy production originating from the mitochondrial damage. Fourth, male Wistar rats were treated for 3 weeks whereas male C57Bl/6J mice were only treated during 5 days. Indeed, the experiments in mice had to be stopped early due to significant weight loss (see “Materials and Methods”). As matter of explanation, the rats weighing 100 g are probably adolescents and mice are not (30 g). Previous work with rodents has shown higher resistance to anticancer drugs in younger animals [38]. Finally, it is unclear why skeletal muscle was unaffected by I-BET-151 treatment and further studies are required to comprehensively analyze the differences in response of these two muscle fiber types.

In conclusion, BET inhibition is a promising new option for cancer management, and recent studies demonstrate a usefulness of BET inhibitors beyond cancer treatment, for instance in the fields of cardiac hypertrophy and heart failure, and of chronic inflammatory diseases. However, the possible context-dependent cardiac side effects of these drugs have to be appreciated. Future studies should focus on the basic mechanisms of the potential cardiovascular toxicities induced by BETi and strategies to minimize these unexpected complications.

## 4. Materials and Methods

### 4.1. In Vivo Studies

Experiments were conducted according to the European Union regulations (Directive 86/609 EEC) for animal experiments and complied with our institution’s guidelines for animal care and handling. The animal facility is licensed by the French Ministry of Agriculture (agreement No. B92-019-01). The Committee on the Ethics of Animal Experiments CEEA26 CAPSud approved the study (Project #13141, approved on 15 June 2017). Dr. Perros supervised all animal experiments (agreement delivered by the French Ministry of Agriculture for animal experiment No. A92-392). All efforts were made to minimize animal suffering.

I-BET-151 (GSK) was given per os in dextrose 5%, DMSO 5%. The 10 mg/kg /day I-BET-151 dose used in rats and the 50 mg/kg/day I-BET-151 dose used in mice are doses commonly used in the literature [23,39]. We used them because it is difficult to extrapolate the doses to be used in rodents from the doses defined for human use due to the different pharmacokinetic and bioavailability profiles among species. Nevertheless, we postulate that the cardiac effects of the 2 mg/kg/day I-BET-151 dose in rats is likely to be similar to that of the human treatment regimen [40].

Male Wistar rats (100 g) were either treated for 3 weeks (W3) or treated for 3 weeks followed by a 3 weeks washout period (W6). Male C57Bl/6J mice (30 g) were only treated during 5 days. Indeed, the experiments in mice had to be stopped early due to significant weight loss. At experiment termination (W3 or W6 for rats and 5 days for mice), animals were anesthetized with 2 L/min O_2_/3% isoflurane (Minerve, Esternay, France) and euthanized by IV injection of KCl (3 M) before tissue sampling. Control animals received only the solvent (dextrose 5%, DMSO 5%) following the same schedule.

The observable side effects of I-BET-151 after 3 weeks treatment (W3) and 3 weeks drug washout (W6) in rats are listed in Table 1.

### 4.2. Echocardiographic Evaluation

Evaluation by trans-thoracic echocardiography (TTE) was performed with a digital ultrasound system (Vivid E9, GE Healthcare, Chicago, IL, USA) by using a high-frequency phased array transducer (12 S-D 4–12 MHz, GE Healthcare). Echocardiographic evaluation procedure was performed under general anesthesia and spontaneous breathing with an Isoflurane Rodent Anesthesia System (Minerve, Esternay, France) (induction: isoflurane 3% at room air; maintenance: isoflurane 2% at room air). Rats were shaved and temperature was controlled during TTE. Time under anesthesia was short enough (less than 15 min) to minimize the effect of isoflurane. TTE examinations were all performed by the same trained operator, avoiding inter-operator variability.

Concerning settings, Pulsed Wave (PW) Doppler interest area was 1.5 mm, gain value was high and frame rate maximal. Data analyses were performed directly or offline with EchoPac Software (GE Medical). All analyses were performed in a blinded fashion: rats’ experimental conditions were unknown by the operator during TTE examination and data interpretation.

Echocardiography were performed at baseline (W0) (before I-BET-151 exposure), W3 and W6 in the same conditions. Measurements were all performed in triplicate (all data were averaged during 5 cardiac cycles) and the following parameters were analyzed:

- In parasternal short axis view: pulmonary artery acceleration time (PAAT), right ventricular ejection time (RVET), cycle length (CL) with heart rate (HR), shape of pulmonary artery outflow pattern, pulmonary artery velocity time integral (VTI Pa), aorta velocity time integral (VTI Aorta), and aorta and Pa valves smallest diameters. Stroke volume (SV) and cardiac output (CO) of left (SV /CO Aorta) and right (SV/CO Pa) outlet tractus were calculated using classical formulas. SV (mL) = VTI × Vessel (Aorta or Pa) surface (cm), and CO (mL/min) = SV × HR. SV and CO are indexed (Si/Ci aorta and Pa) on weight.
- In five cavities view VTI Aorta is analyzed using color and PW dopplers in aorta outlet tractus.
- In four cavities view: RV and LV wall thickness (RVWT, LVWT), RV and LV end-diastolic diameter (RV/LVEDD) and end-systolic diameter (RV/LVESD), interventricular septum shape, TAPSE (Tricuspid Annular Plane Systolic Excursion) in TM Doppler and S tricuspid wave in tissue Doppler imaging coupled with TM.

RV and LV diameter fractional shortening (RV/LV FS) are calculated using the classical formula for each ventricle: LV FS = ((LVEDD − LVESD)/LVEDD) × 100% or RV FS = ((RVEDD − RVESD)/RVEDD) × 100%.

### 4.3. Transmission Electron Microscopy (TEM)

Pieces of papillary muscles (right and left ventricle) and soleus (3 mm^3^) from three animals per group were fixed for 4 h at room temperature (RT) in 2% glutaraldehyde in 0.1 M cacodylate buffer, pH 7.2 at RT. Samples were then contrasted with Oolong Tea Extract (OTE) 0.5% in cacodylate buffer, post-fixed with 1% osmium tetroxide containing 1.5% potassium cyanoferrate, gradually dehydrated in ethanol (30% to 100%) and substituted gradually in mix of propylene oxide-epon and embedded in Epon. (Delta microscopie, Labège, France). Thin sections (70 nm) were collected onto 200 mesh copper grids, and counterstained with lead citrate before observation with a Hitachi HT7700 electron microscope operated at 80 kV (MIMA2-UMR 1313 GABI, Plateau de Microscopie Electronique, Jouy-en-Josas, France). Images were acquired with a charge-coupled device camera AMT–Hitachi (Elexience, Verrière le Buisson, France).

### 4.4. Stereological Analysis

The sections were cut from each tissue block at several randomly selected levels separated by more than 50 μm. Nine pictures were analyzed from two or three hearts for each group. The quality of mitochondria was estimated using ImageJ software. A grid of 2 µm^2^ squares in which each line intersection served as a sample point was generated on each image. According to standard stereological methods, the number of points that overlay mitochondria were counted and the percentage of mitochondria with anomalies (A) or vacuoles (V) was calculated. The sum of mitochondria with anomalies and mitochondria with vacuoles represents the totality of altered mitochondria for a given section and was reported as ‘Total’ (T) in the graphs.

### 4.5. Mitochondrial Functional Assays in Permeabilized Cardiac Ventricular Fibers

Mitochondrial respiration was studied in situ in saponin-permeabilized cardiac muscle fibers using a Clarke electrode as previously described [41]. A protocol was designed to measure oxygen consumption after successive addition of glutamate/malate (10 mM/4 mM), ADP (0.1 mM), ADP (2 mM), pyruvate/succinate (1 mM/15 mM) and amytal (an inhibitor of complex I, 1 mM) to respiration solution (in mM: 2.77 CaK2 ethyleneglycol tetraacetic acid (EGTA), 7.23 K^2+^EGTA [100 nM free Ca^2+^], 6.56 MgCl_2_ [1 mM free Mg^2+^], 20 taurine, 0.5 dithiothreitol (DTT), 50 K-methane sulfonate [160 mM ionic strength], and 20 imidazole. pH 7.1) at 23 °C. Rates of respiration are given in µmoles O_2_/min/g dry weight.

### 4.6. Enzyme Activity

Frozen tissue samples were weighed and homogenized (Bertin Precellys 24) in ice-cold buffer (50 mg/mL) containing 4-(2-hydroxyethyl)-1-piperazineethanesulfonic acid (HEPES) 5 mM (pH 8.7), EGTA 1 mM, DTT 1 mM and 0.1% Triton X-100. Activity of citrate synthase (CS) and cytochrome oxidase (COX) were determined using standard spectrophotometric assays [42].

### 4.7. Stereological Analysis

Results are expressed as mean ± SEM. Statistical differences were analyzed using multifactorial ANOVA; Newman-Keuls post-hoc tests were used to identify significant differences between means.

## Figures and Tables

**Table 1:** Proportion of altered mitochondria amongst total mitochondrial population. Altered mitochondria were mitochondria with anomalies (A) or vacuoles (V). The sum of mitochondria with anomalies and mitochondria with vacuoles represents the totality of altered mitochondria (Total). Statistical analysis: multifactorial ANOVA. ¶—*p* ≤ 0.05 for the dose effect; §—*p* ≤ 0.05 for the washout effect; ¥—*p* ≤ 0.05 for the ventricle effect; i—*p* ≤ 0.05 for the interaction effect. Post hoc Newman–Keuls test: * *p* ≤ 0.05, ** *p* ≤ 0.01, *** *p* ≤ 0.001 for difference versus the control group (at a given time and for a given ventricle), ¤—*p* ≤ 0.05 for difference between IBET 2 mg/kg/day and IBET 10 mg/kg/day (at a given time and for a given ventricle), $$—*p* ≤ 0.01 for difference between W3 and W6 (for a given dose and a given ventricle), £—*p* ≤ 0.05 for difference between right ventricle and left ventricle (at a given time).

Mitochondrial Alterations	Groups	Statistical Analysis								
		W3-RV	W6-RV	W3-LV	W6-LV	ANOVA	W3-RV	W6-RV	W3-LV	W6-LV
Anomalous	Control	5.2 ± 2	3.2 ± 2.2	5.1 ± 2.7	2 ± 1.6	¶, §, i	-	-	-	-
	I-BET 2 mg/kg/day	25 ± 4.3	14.5 ± 6.2	11.6 ± 3.8	13.1 ± 4		*	-	-	-
	I-BET 10 mg/kg/day	30.7 ± 6	12.2 ± 3.4	31.8 ± 5.2	26.8 ± 7.1		**	-	**, ¤	**
Vacuolized	Control	2.1 ± 1	1.7 ± 1.3	2.1 ± 1.2	0 ± 0	¶, ¥	-	-	-	-
	I-BET 2 mg/kg/day	17.7 ± 5.3	14.3 ± 6.2	28.8 ± 9	23.4 ± 5.2		-	-	*	*
	I-BET 10 mg/kg/day	31 ± 9.9	4.8 ± 4	29.4 ± 11.1	34.1 ± 8.2		*	-	*	*
Total (A + V)	Control	7.2 ± 2.8	4.9 ± 2.2	7.2 ± 3	2 ± 1.6	¶, §, i	-	-	-	-
	I-BET 2 mg/kg/day	42.8 ± 8.6	28.7 ± 10.7	40.4 ± 9	36.5 ± 7.2		*	-	*	*
	I-BET 10 mg/kg/day	61.7 ± 10.9	17 ± 4.2	61.3 ± 8.4	60.9 ± 10.7		***	$$	***	***, £

## Abbreviations

AMI	acute myocardial infarction
BRD	bromodomain
BET	bromodomain and extra-terminal domain
BETi	bromodomain and extra-terminal domain inhibitor
CS	citrate synthase
COX	cytochrome oxidase
P-TEFb	positive transcription elongation factor
TEM	transmission electron microscopy
HF	heart failure
RV	right ventricle
LV	left ventricle
VTI	velocity-time-integral
RVFS	right ventricular fractional shortening
LVFS	left ventricular fractional shortening
TAC	transverse aortic constriction
